# Development of a Continuous Biogas Pressure Measurement Device with Applications in Batch Anaerobic Digestion Tests

**DOI:** 10.1021/acsomega.4c11331

**Published:** 2025-08-12

**Authors:** Andrea Pérez-Vidal, Leonardo Antonio Bermeo Varón, Lina Mariana Rodríguez-Jiménez, Sonia María Bolaños-Muñoz, Yordy Mario Lemos-Valencia, Jorge Antonio Silva-Leal, Patricia Torres-Lozada

**Affiliations:** a Faculty of Engineering, School of Natural Resources and Environmental Engineering, 28006 Universidad del Valle, 13th Street, 100-00, Cali 760032, Colombia; b Faculty of Engineering, School of Electrical and Electronics Engineering, 28006 Universidad del Valle, 13th Street, 100-00, Cali 760032, Colombia; c Faculty of Engineering, 28027 Universidad Santiago de Cali, Fifth Street, 62-00, Cali 760035, Colombia; d Faculty of Engineering, 249001 Fundación Universitaria Católica Lumen Gentium, 122th Avenue, 12-459, Cali 760008, Colombia

## Abstract

The manual manometric (MM) method is widely used in batch anaerobic digestion tests, such as the biochemical methane potential (BMP) and the specific methanogenic activity (SMA), but it can cause inaccuracies due to biogas loss during measurements. This study presents an IoT-based biogas pressure measurement device developed with an Arduino microcontroller to improve accuracy and reliability in batch tests. The device supports four reactors and was tested in 250 mL glass vessels with varying headspace (20 and 50%) and substrate/inoculum ratios (0.5 and 0.2). Two tests were performed: one with glucose (11 days) and another with food waste (60 days). A third 30 h test compared the IoT device to the MM method under identical conditions. All tests used inoculum from a septic tank. The results showed that the MM method underestimates biogas production, with an average biogas loss of 50.7 ± 12.9 mbar per measurement. These losses increased at higher reactor pressures. Implementing IoT-based monitoring in BMP or SMA testing improves measurement accuracy, enables early failure detection, and facilitates efficient data management. In addition, the IoT device allows real-time observation of early biogas production dynamics, including acidogenic and methanogenic activity, as well as the detection of potential methanogenesis inhibition.

## Graphical abstract

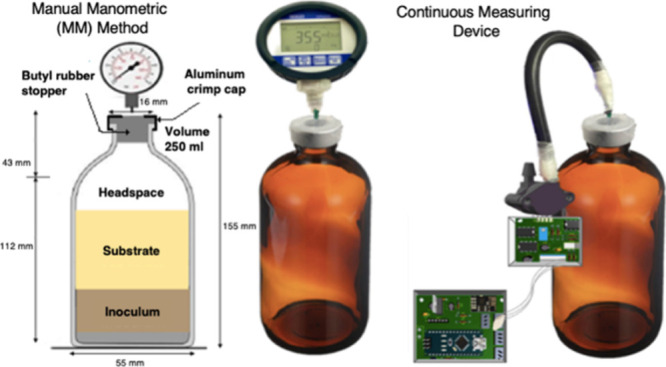

## 1 Introduction

Anaerobic digestion (AD) is a biochemical process in which microorganisms interact in the absence of oxygen to decompose organic matter into simpler molecules.^1^ It is used in the treatment of different organic substrates such as wastewater, food waste, anaerobic sludges, and energetic substrates as a potential alternative for energy generation.^2^ The biogas produced by AD is mainly composed of methane (approximately 70%).^3^ In addition, in the fermentation process hydrogen can be obtained from the decomposition of carbohydrates present in organic substrates,^4^ with methane and hydrogen being gases with high energy potential.

During anaerobic biodegradability, inhibition, and toxicity studies, batch tests have been widely used with multiple applications to evaluate the energy production potential of a substrate and biogas yield, selecting the inoculum and identifying the adaptation or inhibition of microorganisms. Among batch tests, specific methanogenic activity (SMA)^3,5^ and biochemical methane potential (BMP) can be highlighted.^6^

Generally, there is no standardized or mandatory procedure for evaluating and interpreting experimental batch test results beyond the termination criterion and the standardization of the gas produced.^7^ The biogas produced is confined within the bioreactor, generating a proportional excessive pressure that can be gauged using a differential manometer.^8–10^ Manometric or volumetric methods can be used to perform batch testing.^7^ A variety of techniques can be used to measure biogas, including volumetric methods (typically NaOH solution displacement), manometry (determining pressure variation via transducers), gas chromatographic methods with flame ionization detectors or thermal conductivity detectors^6,9–11^ and gravimetric methods based on the measurement of mass loss as a result of biogas removal.^12^

Although the manometric method is one of the most widely used methods in laboratories worldwide due to its low technical requirements, the influence of variables such as headspace, measurement frequency, amount of needle punctures and upper pressure release,^12–14^ combined with potential human errors, can lead to an underestimation of BMP values.

One of the main challenges in accurately quantifying total gas production arises from the solubility of carbon dioxide (CO_2_) in the digestion liquid, as it affects the pH, pressure, headspace, temperature, and thermodynamic equilibrium established between CO_2_ and calcium/magnesium carbonates/bicarbonates.^15^ An increasing amount of CO_2_ dissolves in the medium as its partial pressure increases in the headspace. As a result, according to Henry’s law,^13^ releasing less gas during pressure measurement could potentially affect pH and alter microbial activity, leading to partial inhibition of the process.^14,16^

Although increased in pressure during anaerobic digestion can reduce the yield of the process due to the decrease in pH caused by increased CO_2_ solubilization, pressurized anaerobic digestion (PDA) has garnered growing interest because it improves biogas production by increasing the methane fraction while decreasing CO_2_.^17^

Some results indicate that anaerobic reactors can operate continuously at high pressures, providing a viable alternative that reduces the subsequent effort required for biogas purification.^18^ However, increasing pressure can also lead to a higher concentration of volatile fatty acids (VFA) and their accumulation, which is directly related to the characteristics of the feedstock.^17^ This is because the amount of dissolved CO_2_ will depend on the initial chemical oxygen demand (COD) content, assuming constant operating parameters such as temperature, pressure, and pH.^19^

In light of the operational challenges and variable gas compositions observed under different digestion conditions, reliable and continuous monitoring of methane production has become increasingly important. The development of automated systems for quantifying methane (CH_4_) can be attributed to advances in technology and the increasing interest of researchers in online monitoring of AD processes.^20^ These systems include Oxitop (based on the manometric method), AMPTS II (based on the volumetric method), Envital kit (based on a fluorescent redox indicator) and spectroscopy techniques that predict the BMP value using spectral data.^8^

These systems enabled the acquisition of high-quality biogas pressure data, thereby facilitating a comparison of the results. However, their implementation involves resource investment, which laboratories and researchers in developing countries generally lack. Furthermore, some of these systems are still in the early development phase, requiring validation.^21,22^

In this context, this study presents the development of a continuous biogas pressure measurement device with an affordable, accessible, and reproducible design aimed at laboratories and researchers working in the field of AD. Existing manometric methods, although widely used, are prone to gas loss and measurement errors, especially in low-resource settings where advanced systems are often unaffordable or unavailable. The proposed system integrates Internet of Things (IoT) technology^23^ and uses Arduino, ESP8266–01, and the MPX2050 CASE 344 pressure sensors to enable real-time data collection from batch tests, such as the BMP test. The novelty of this work lies in the implementation of a low-cost, open-source hardware platform that enables continuous and reliable monitoring of biogas pressure, offering a scalable alternative to conventional manual techniques and contributing to improved data quality, early detection of process failure, and greater adoption in developing regions.

## 2 Methodology

### 2.1 Development of a Continuous Biogas Pressure Measurement Device

The device in the embedded system includes a microprocessor based on Arduino and ESP32, along with specific functions programmed for continuous pressure measurement. The electronic design of the system is described below, including hardware and software specifications.

#### 2.1.1 Electronic Design

The main devices used in the pressure measurement system are shown in Figure 1. The design incorporated an Arduino board that included the DS3231 RTC module and SD read/write module. It functions as a storage device in the event of a Wi-Fi connection failure. It is a Wi-Fi module (ESP8266–01) with a low drop-out voltage regulator (LM1117t), which guarantees a voltage of 3.3 V required by the ESP-01 microcontroller. Additionally, the Arduino board’s acquisition circuit comprises an amplifier stage, offset elimination, and MPX2050 pressure sensor.

**Figure 1:**
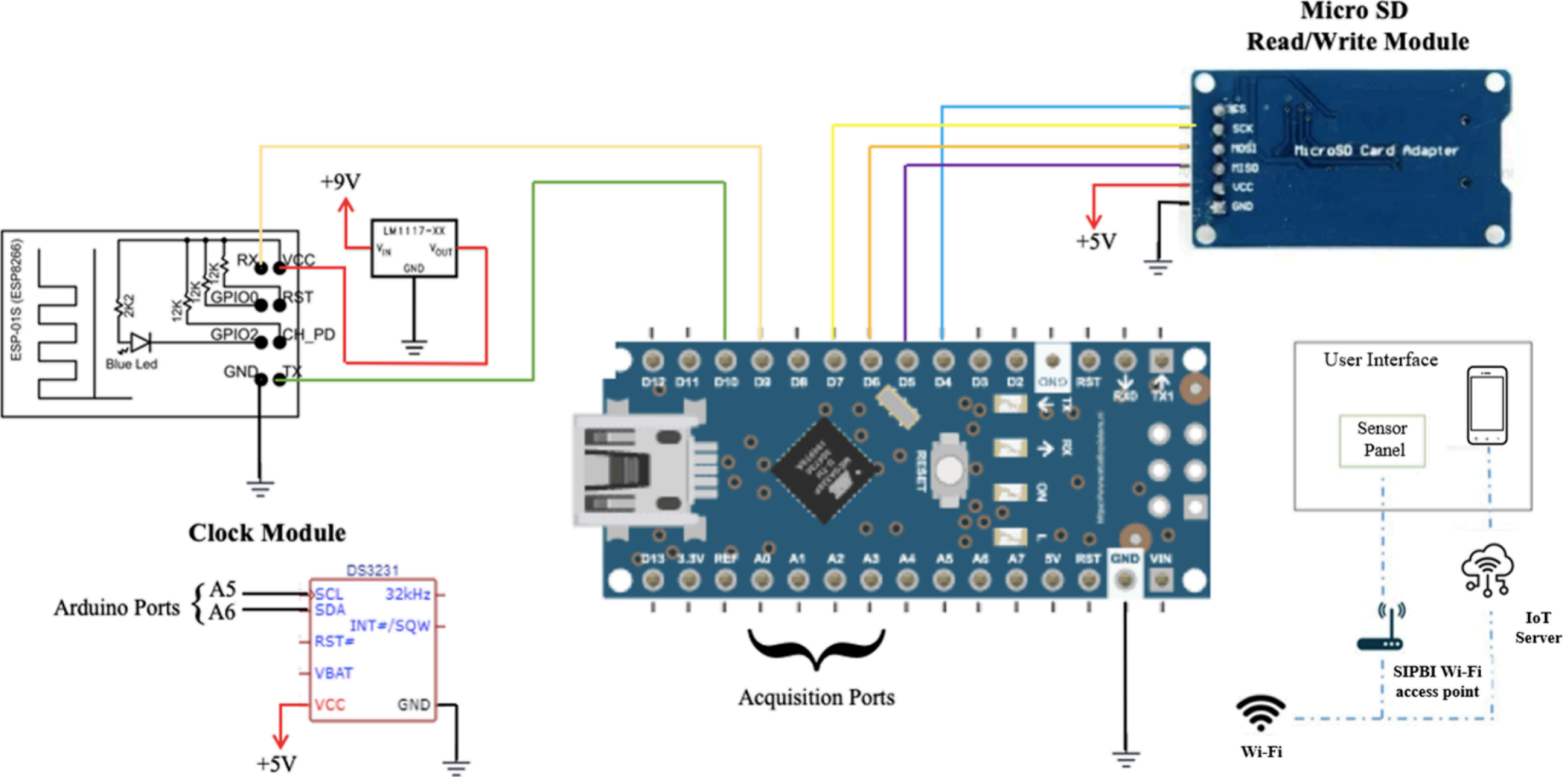
Electronic design.

The characteristics of the sensor are listed in Table 1.^24^ All devices were placed on a printed circuit board with a 9 V power supply.

**Table 1:** Characteristics of the Sensor^a^

characteristics	MPX2050
pressure range (mbar)	0–500
power supply (V)	5–16
current supply (mA)	6
operation temperature (°C)	–40 a 125
linearity	±0.25%V_FSS_
temperature compensation	yes
sensibility	0.8 (mV/Kpa)
hysteresis	±0.1%V_FSS_
dimension (cm × cm × cm)	2.985 × 2.934 × 1.105

a Source: Adapted from Freescale.^24^

The acquisition circuit receives a positive power supply from an Arduino board and a negative power supply from an ICL7660 voltage converter. This circuit is divided into two stages: *i*. amplifier stage, and *ii.* offset elimination. The first stage used an AD620 instrumentation amplifier with a gain of 215, whereas the second stage used an LM358 amplifier. Figures 2 and 3 show details of the schematic design of the system.

**Figure 2:**
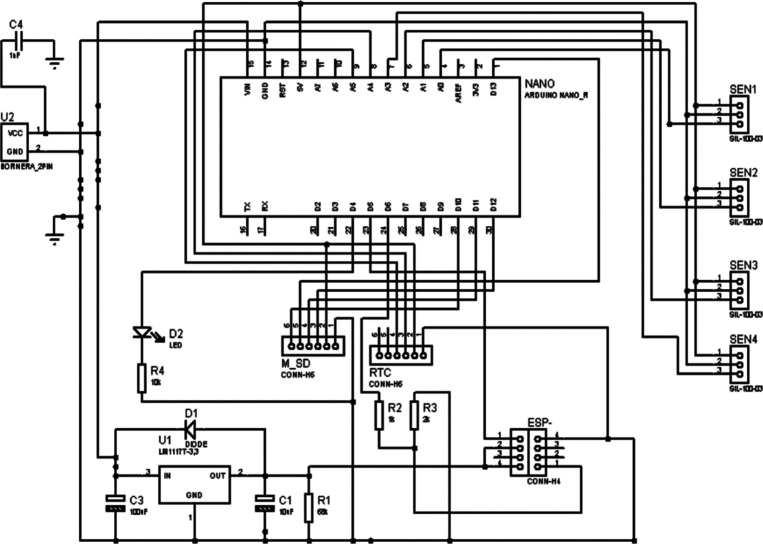
Schematic design of Arduino board.

**Figure 3:**
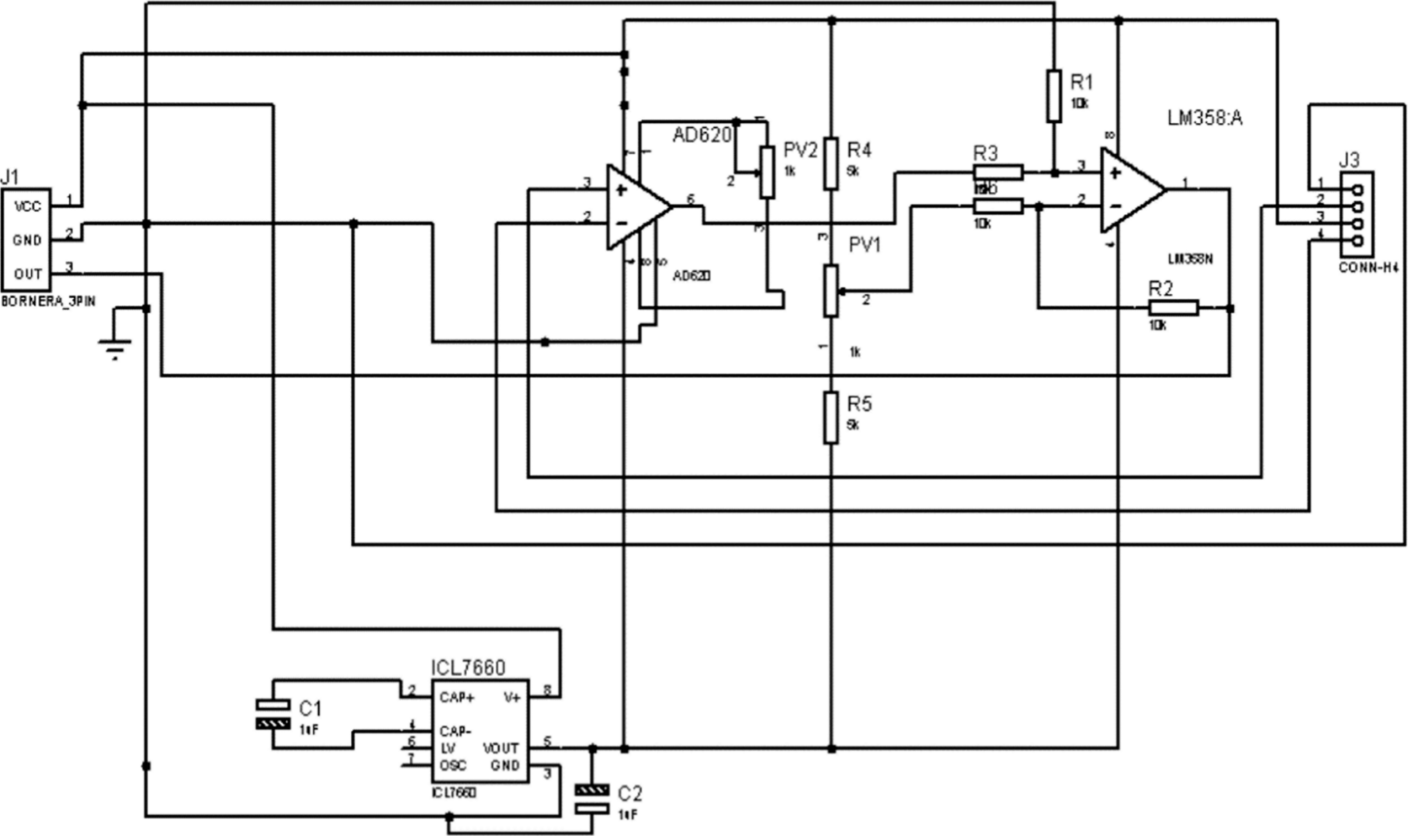
Schematic design of signal conditioned and power supply

The device supported by IoT technology enables remote display, storage, and monitoring of the biogas pressure signal during batch testing. This device has a resolution of 1 mbar, with a maximum allowed error of 2.5 mbar and uncertainties of 7.6 mbar. The system is housed in an acrylic box for component protection and features four ports to connect an acquisition board to monitor four reactors, including a pressure sensor, an acquisition board, a processing board housing the microprocessor, a Wi-Fi module and the remaining components. The board accommodated a maximum of four pressure sensors.

The code for acquiring and processing the pressure measurements was developed on an Arduino board and Esp82266–01 Wi-Fi module, available at https://github.com/lebermeo/Continuous_Biogas_Pressre_Measurement_Device. The Arduino board features the following functions: (i) obtaining the date and time, (ii) data logging, (iii) data processing and digitization, and (iv) hardware control. The ESP8266–01 module exhibits the following functions: (i) connection to the IoT platform, (ii) Wi-Fi access point configuration, (iii) server start-up, (iv) user interface management, and (v) Wi-Fi connection credentials management. Serial communication is facilitated between the two modules.

#### 2.1.2 Configuration of the System

The device was adapted for the BMP test setup, as well as operational variables such as temperature and headspace, were considered when selecting the sensor type and materials for the device design.^1,16^ Amber bottles of 250 mL were utilized as reactors, with a 20 and 50% headspace (50 to 125 mL),^14,16^ a substrate to inoculum ratio (S/I) of 0.5 and 0.2 g TVS substrate/g TVS inoculum,^5,10^ and a temperature controlled at 35.0 °C ± 0.1 °C using a JPS-LAB refrigerated incubator. The bottles were sealed with butyl rubber stoppers and aluminum crimp caps to avoid pressure leakage.

Following the recommendation of Pabón-Pereira and colleagues,^25^ the application of NaOH beads for the capture of CO_2_ was removed to reduce the risk of AD inhibition due to the possibility of NaOH contact with the substrate and subsequent increase in pH.

Figure 4(a) shows the reactor configuration, indicating the procedure for performing pressure measurements using a manometer.^14^ Figure 4(b) presents the continuous measuring device (S), comprising three components: *i*. pressure sensor, *ii.* acquisition board, and *iii.* processing board. The reactors were operated in batches without stirring.

**Figure 4:**
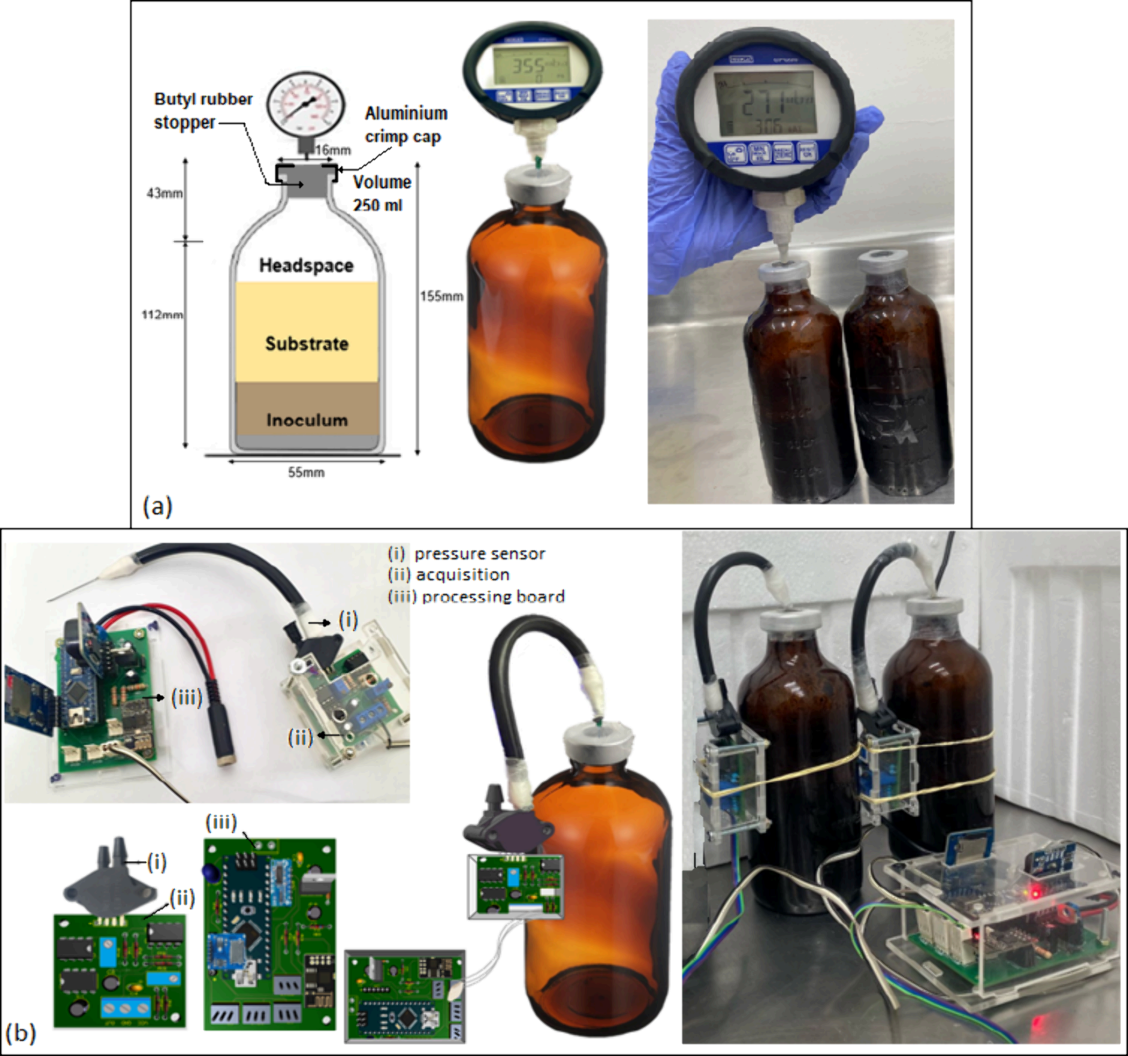
BMP assembly utilizing: (a) manual manometric (MM) method, and (b) continuous measuring device (S).

The device was connected to the reactor through a hose that serves as a conduit to guide the biogas produced from AD to the sensor. This was accomplished by affixing a needle to the hose and inserting it into the butyl rubber stopper, thereby reinforcing the connection at both ends of the hose with Teflon tape and parafilm to prevent potential pressure leaks. The sensor plate was secured to the bottle using two elastic bands to prevent movement or misalignment of the components.

### 2.2 Functional Testing of the Device

The device was evaluated through two batch tests (11 and 60 days) under a BPM test setup to validate its functionality and assess its performance in both short- and long-run conditions. All tests were carried out in duplicate and at a temperature of 35 °C.

#### 2.2.1 Short-Run Test with Glucose Substrate

The experiment evaluated four systems consisting of two assemblies equipped with continuous measurement devices, denoted Sensor 1 (S1) and Sensor 2 (S2), and two assemblies equipped with manometers, denoted as MM1 and MM2. The biogas pressure was designated as the response variable.

An inoculum consisting of anaerobic sludge from a septic tank treating domestic wastewater was added to each bottle at a total volatile solid (TVS) concentration of 1.5 g/L.^9^ The inoculum had a TVS/TS of 0.77 (TVS: 24822 ± 717 mg/L and TS: 32049.5 ± 720 mg/L). A substrate mixture of glucose and a solution of macro and micronutrients was utilized at a chemical oxygen demand (COD) concentration of 709.3 mg/L and a TVS of 0.75 g/L (VDI 2006). A substrate/inoculum (S/I) ratio of 0.5 gVS__substrate_*gVS__inoculum_was maintained under these conditions. Each bottle was sealed with a headspace of 20%.

Although AD can occur in a pH range of approximately 6.0 to 8.5, slightly acidic conditions predominate in the pH range of 6.0 to 6.5, which can inhibit the methanogenesis phase.^26^ The pH of the substrate was adjusted with sodium bicarbonate (NaHCO_3_) to a value of 6.5 in this batch test to maintain a slightly acidic pH in the mixture. This adjustment aimed to validate one of the sensor’s capabilities, which involves early detection of operational issues, allowing proactive decision-making to identify and address potential inhibitions or instability in the AD process from the outset.^27^

The pH of the substrate (SM-500-H+), COD (SM 5220), and total alkalinity (SM-2320B) were measured prior to the BMP test.^28^ In addition to the buffer index in according to Pérez and Torres,^29^ these parameters were also measured at the end of the test (IB = ml of acid used for titration between pH 5.75 and 4.3/ml of acid used for titration up to pH 4.3).

Biogas measurements were conducted twice daily from Monday to Friday, using the MM method. However, the initial pressure reading using the MM method was recorded 40 h after the start time of the experiment (Saturday, 4:00 pm). In contrast, sensor-based systems recorded continuous measurements every hour.

#### 2.2.2 Long-Run Test with Food Waste Substrate

The same experimental setup used in the short-run test was applied replacing the substrate with food waste (FW) having a particle size of 6–8 mm.^9,11,30^ The substrate-to-inoculum ratio was adjusted to 0.2 gVS__substrate_*gVS__inoculum_, by adding 26 gVS/L of inoculum and 5.2 gVS/L of FW. The reactor pH was adjusted to 7.2 by adding a bicarbonate solution. A headspace of 50% was used.

The FW was composed of 51.5% carbohydrates, 0.99% lipids, and 3.4% proteins. Its physicochemical characteristics included a pH of 5.17, moisture content of 80.3%, COD of 263792 mg/L, total organic carbon (TOC) of 48.1%, and total alkalinity of 2200 mg CaCO_3_/L. The total solids (TS) and total volatile solids (TVS) contents were 22% and 15%, respectively. Based on initial indications of biogas loss observed during the short-run test, the measurement frequency using the (MM) method was reduced to three times per week. Meanwhile, sensor-based systems continued to record continuous hourly measurements. The BPM values (mL CH_4_/g SV) were calculated according to the VDI 4630 methodology,^9,11,30^ and biogas composition was analyzed on the eighth day of the experiment using gas chromatography.

#### 2.2.3 Simultaneous Pressure Measurement Test

A supplementary batch test was performed to quantify the gas losses that occurred during manometer measurements. A manometer was implemented in two assemblies with sensors S1 and S2 to simultaneously measure pressure every 5 h for a duration of 30 h.

Each bottle was inoculated with the same anaerobic sludge from a septic tank used in previous experiments, at a concentration of 1.5 g TVS/L, ensuring an S/I ratio of 0.5. Shredded food waste with a particle size of 6–8 mm was selected as the substrate due to its higher biogas generation potential. A headspace of 20% was maintained in each reactor, and the substrate was added to achieve a TVS concentration of 0.75 g/L and a COD of 7210 mg/L.

Pressure measurements were initiated 2 days after the bottles were sealed to ensure adequate accumulation of biogas in the headspace.^16^ Specifically, six measurements were performed between 48 and 78 h after the start of the batch test.

### 2.3 Statistical Analysis

The evaluation of the results obtained from the methods employed was performed using statistical analysis, specifically using a *t* test for independent samples. This test was instrumental in determining whether there were significant differences between the means of the two compared groups. In addition, a comprehensive variance analysis was performed to further assess the effectiveness of both methods, using the R software (version 2022.02.0–443) for the cases. To enhance the interpretation of the results, a confidence interval was also calculated, providing an estimated range that is expected to encompass the true values of the measured variables with a specified level of certainty. This multifaceted approach not only strengthens the validity of the findings, but also offers a clearer understanding of the implications of the data in the context of the study.

## 3 Results and Discussion

Figure 5 shows the pressure data reported during the short-run glucose substrate test over 11.3 days (270 h) of the AD process. Measurements were obtained from two systems using sensors (S1 and S2) and two manometer-based systems (MM1 and MM2). An increase in biogas pressure was observed during the initial hours. However, around 50 h, there was a decrease in pressure for the manometer and sensor-based setups. The observed decrease in pressure for both methods can be attributed to the mixture maintaining a slightly acidic pH (6.5 units) at the start of the test. This, combined with the fact that glucose is an easily acidified substrate, suggests the onset of an inhibitory process caused by acidification during the test.^31^

**Figure 5:**
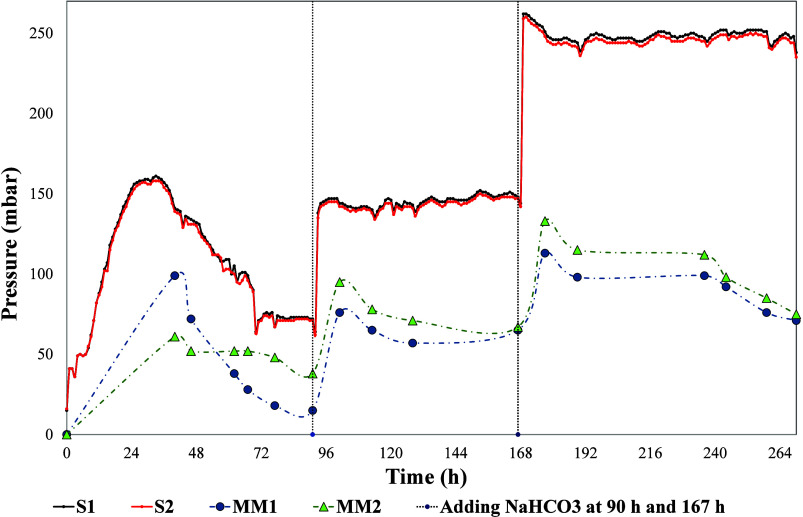
Biogas pressure measured in the short-run glucose substrate test, using both S and MM methods.

This decrease may be attributed to the transition of CO_2_ to the aqueous phase due to an increase in its partial pressure (PCO_2_) within the free space of the reactor during the acidogenesis and acetogenesis stages, leading to the production of carbonic acid (H_2_CO_3_). This situation probably results in the inactivation of acetogenic bacteria, leading to the accumulation of VFA and inhibition of the AD process.^27^ Therefore, higher headspace values are more advisable, between 50 and 75%.^13,14^

Sodium bicarbonate solution (10 mL) was immediately injected into each bottle (reactor) of both setups (sensor and manometer) at the 90-h mark of the test. Both methods displayed an immediate response, characterized by an increase in biogas pressure. This is due to the reaction of sodium bicarbonate with acids, such as acetic acid, which are generated during the process. This reaction allows CO_2_ trapped in the aqueous phase to be released into biogas,^32^ resulting in an immediate and rapid increase in biogas pressure, possibly due to a decrease in headspace volume and an increase in CO_2_ concentration in the gas phase.

The identical procedure of injecting 10 mL of sodium bicarbonate into each bottle was repeated after 167 h, resulting in an increase in pressure for both methods. Subsequently, the curves displayed a more constant trend, probably indicating that the process had stabilized. A comparison of the manometer results with those of the continuous pressure measurement device reveals that the pressures reported by S1 and S2 are very similar, with a correlation of 0.99 and a p-value of 0.1863, indicating that there were no significant differences.

Concerning MM1 and MM2, the correlation was lower (0.85) and the p-value was higher (0.5522), indicating that there were no significant differences. However, the recorded pressure was lower than that of the continuous measured pressure. We obtained a correlation of 0.66 and a p-value of 0.0055 when comparing the two methods. This indicates that, although the measurements are correlated, they have significant differences, as the p-value is less than 0.05.

In general, the test conducted with MM produced less biogas than the test conducted with the sensors. This may be attributed to the fact that previous research has indicated that gas production using the MM method can be affected by variables such as the number of needle punctures, upper pressure, and release frequency.^13,14^ Consistent with the findings of other automated methods described in the literature, continuous pressure measurement demonstrates^21,33^ its potential to reduce errors caused by human interference.

Table 2 presents the process control variables measured at the beginning and end of the test. COD removal was observed in all systems that were evaluated, with removal efficiencies ranging from 78.8 to 84.4%. The increase in pH at the end of the BMP test was due to the addition of sodium bicarbonate at 90 and 127 h of the test, which confirmed that buffer capacity was achieved, and the process stabilized. The buffer index had values below 0.2, indicating that the reactors were underfed and not acidified.^29^

**Table 2:** Control Parameters Measured at Beginning and End of the Test

	pH		COD (mg/L)		total alkalinity (mgCaCO_3_/L)		buffer index
system	initial	final	initial	final	initial	final	final
MM1	6.51	7.62	709.3	110.53	60	2350	0.14
MM2	6.57	7.49		150.46		2300	0.17
S1	6.50	7.55		146.21		2270	0.12
S2	6.51	7.67		116.89		2350	0.14

Glucose is an easily degradable substrate that tends to accelerate acidogenesis.^34^ This can result in rapid accumulation of CO_2_ in the reactor headspace, which in turn increases the partial pressure of the gas and causes CO_2_ to transition to the aqueous phase according to Henry’s law.^13^ As a result, reductions in biogas pressure were observed during test durations of 90 and 127 h. Regardless of the variations in pressure caused by the biogas composition within the reactor, the sensor-based systems responded appropriately and enabled data recording at an hourly frequency. This provides accuracy and reliability for this method, as well as early detection of potential inhibitions or instability in batch anaerobic digestion tests.

Figure 6 presents the sample averages for each measurement process and the confidence interval (CI = 95%) to validate the influence of the biogas production monitoring method. It was observed that the test conducted using the continuous pressure measurement process had a smaller confidence interval than that observed for the MM process. This is due to the reduced variability between individual systems in the sensor method compared to the MM method. This behavior is consistent with that reported in the literature for other automated methods,^21,33^ highlighting their potential for reducing errors due to human interference.

**Figure 6:**
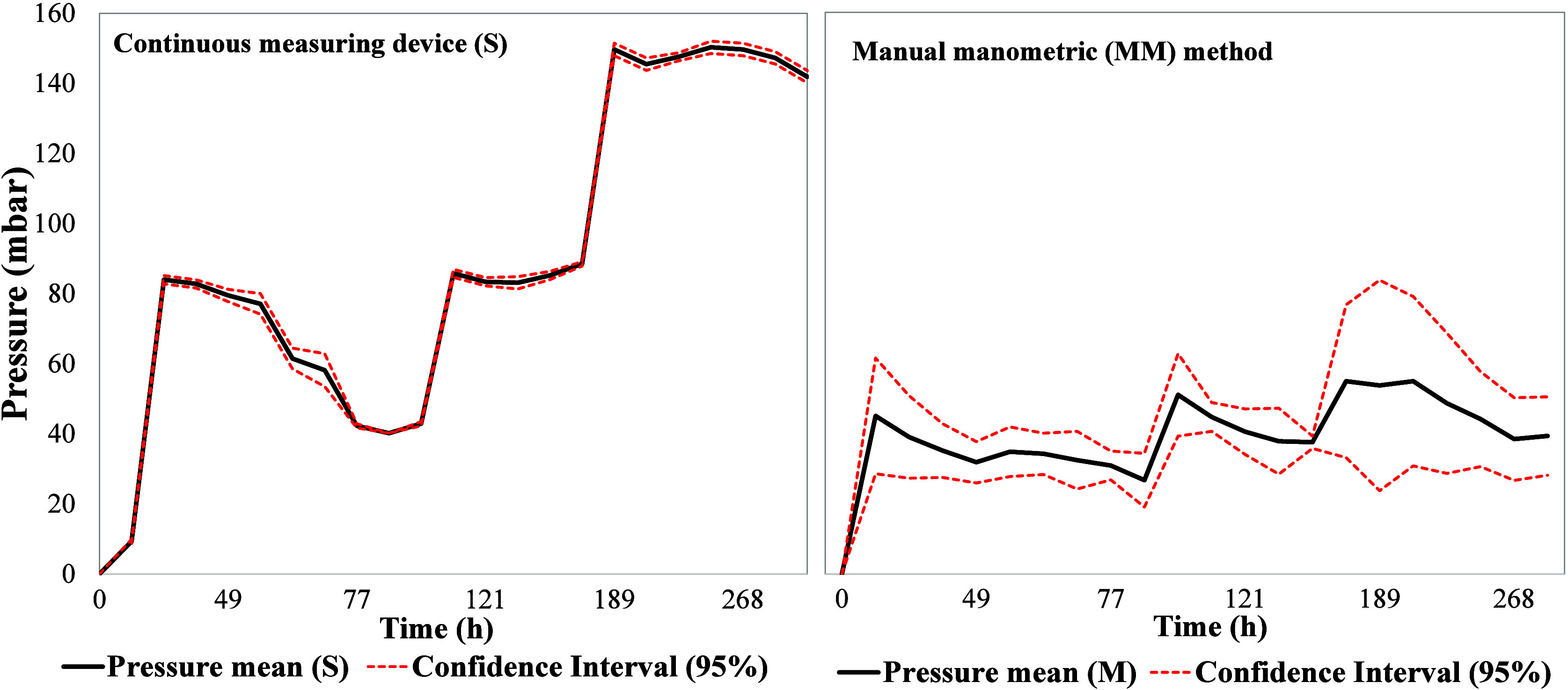
Sample confidence interval (95%) using both methods.

Figure 7 shows the data recorded during the long-run test with a Food Waste Substrate over 60 days. The replicates were averaged to generate the curves, given that no statistically significant differences were observed among them (p-value >0.05). The horizontal arrows indicate the days on which the reactors were vented after reaching 500 mbar, in order to prevent overpressures exceeding the recommended range for BMP tests (100–600 mbar),^14,16,30^ as well as the sensor measurement limit (see Table 1). At these points, although both the MM method and the pressure sensor resumed the measurement from a lower pressure value after venting, the amount vented was added to the subsequent data point. This adjustment ensured that the recorded and plotted values reflected the total pressure that would have been reached without venting.

**Figure 7:**
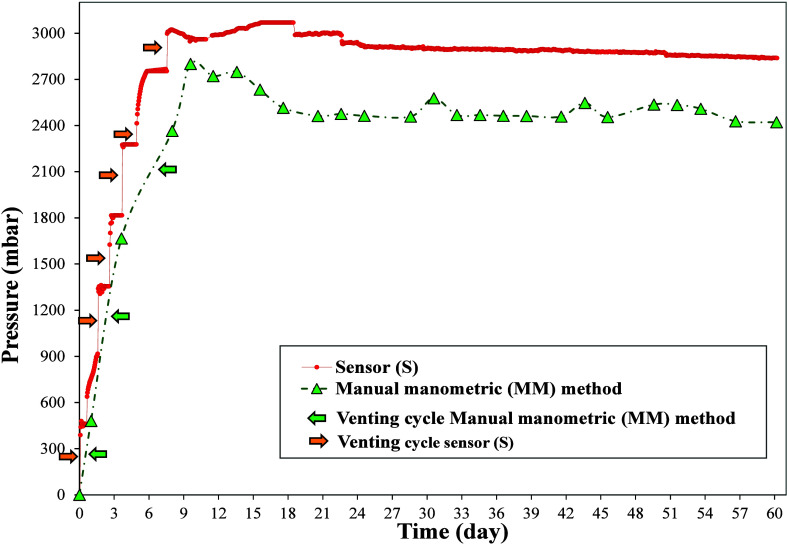
Biogas pressure measured in the long-run test with FW, using both S and MM methods.

Similar to the short-term test, pressure differences between the two methods were also observed in the long term data. The MM method consistently showed lower values, primarily due to biogas losses during punctures, resulting in an average pressure drop of 410 ± 106.7 mbar per puncture. The greatest pressure losses occurred during the first 8 days (up to 651 mbar), a period typically associated with the early stages of anaerobic digestion, during which the elevated presence of CO_2_ is likely linked to initial acidogenic activity and partial degradation of readily available substrates. Gas chromatography analysis confirmed the predominant presence of CO_2_, with results showing 72.10% CO_2_ and 4.33% methane.

The accumulation of these losses over time can affect the final results, as pressure buildup is a key factor in the BMP methodology. Individual losses gradually shift the curve, leading to a deviation from the actual conditions. The BMP value obtained using the MM method was 349.9 mL CH_4_/g VS, while the sensor system yielded a value of 481.3 mL CH_4_/g VS, representing a difference of approximately 27.3%.

Although the final BMP value may not differ significantly, the use of the manometric method can lead to slight underestimations due to cumulative pressure losses. As noted by Amodeo et al.,^12^ even small differences between the manometric method and other approaches may still be important when aiming for the most accurate BMP measurements.

Figure 8 shows the comparative simultaneous measurement test with both methods (MM and S) (i.e., continuously measuring and with the manometer in the same reactor). The initial pressure was observed to be approximately 350 mbar, exceeding the values recorded in the previous batch test. This difference is attributed to the type of substrate used, as the COD value of glucose is 97% lower than that of food waste, resulting in significantly higher estimated biogas production.^9,35^

**Figure 8:**
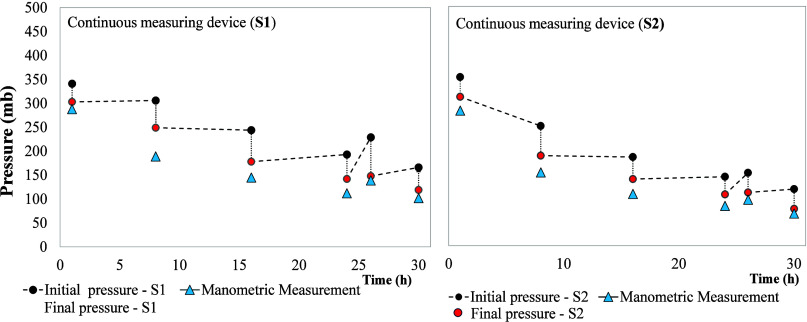
Simultaneous pressure measurements test.

The pressure recorded by the sensors before and after the measurement with the manometer dropped abruptly. This was due to the insertion of the manometer needle into the bottle stopper, which allowed biogas to escape. The accumulation of biogas loss was reflected in subsequent measurements. Hence, the differences are attributed to the manometer pressure measurement protocol rather than erroneous measurement by the sensor system.

These tests allowed us to establish that there was a loss of biogas when the pressure was measured with a manometer. The loss of biogas pressure in these tests was recorded to be 50.7 ± 12.9 mbar at each measurement over a period of 30 h. It is important to note that these losses became more significant at the end of the test.

The cumulative loss of biogas during batch tests can underestimate the maximum biogas potential. Consequently, the results of this test indicate that the measurement frequency of the MM method is a highly significant and influential variable in the final results of tests such as BPM. A higher measurement frequency corresponded to a greater loss of biogas and an increased risk of plug damage.^12,14,16^

The initial batch test in this study was performed at an average frequency of 1 to 2 times per day, followed by a second test with measurements taken every 3 days, and a final test with measurements every 5 h. Biogas loss was evident in all three tests during each MM measurement. Given the widespread use of the manometric method, due to its simplicity and low cost,^12^ it is advisable to consider less frequent measurement intervals (e.g., every 2 or 3 days) to reduce biogas losses and avoid underestimating the maximum biogas potential.

Another factor to consider is the risk of excessive pressure within the reactor, which can lead to dissolution of CO_2_ in the aqueous phase, resulting in a subsequent decrease in pH.^17^ Therefore, it is also important to consider higher percentages of headspace (e.g., 50%), as recommended by Hafner and Astals,^14^ and to consider the purging of biogas for headspace release, as advised by Himanshu and colleagues,^13^ Hafner and Astals,^14^ Valero and partners.^16^ The sensation of inflated plug sensation was noticeable during the experimental observations when there was a substantial accumulation of gas, which could provide qualitative insights into the definition of the purging frequency.

Concerning the device, we highlight that our work presents distinct advantages, including enhanced integration with IoT capabilities using Wi-Fi modules. This integration facilitates improved data accessibility and real-time monitoring, both of which are essential in today’s connected environments. We believe that these features, along with our device’s reliability and accuracy, position it favorably within the current landscape of sensor technologies. In addition, the device can be adapted for use in high-pressure anaerobic reactors, provided that a sensor capable of measuring the required pressure ranges is utilized. With the necessary adjustments, it is simply a matter of replacing the existing sensor with one with a higher range. It should be noted that, although a sensor with a higher measurement range could be used, reaching overpressure is not recommended in BMP and SMA assays. Excessive pressure can cause CO_2_ dissolution, leading to a reduction in pH and negatively impacting the performance of anaerobic digestion.^17^

Finally, from an economic perspective, the utilization of a continuous measurement device is feasible in developing countries and can serve as a mechanism for monitoring or validating the MM method. The cost of the continuous measurement device equipped with four sensors was estimated at 195.0 USD.

## 4 Conclusions

The IoT device developed in this study demonstrates a considerable ability to eliminate uncertainties caused by human error and achieve better precision of the pressure variable than the MM method. The MM method tends to underestimate pressure values due to biogas losses during each measurement, with an average loss of 50.7 ± 12.9 mbar at each measurement over a period of 30 h. In the long term test, pressure losses of approximately 410 ± 106.7 mbar per puncture were recorded, resulting in a 27.3% difference in the BPM calculated between the MM method and the sensor system, with the sensor yielding higher values. These losses are mainly attributed to the higher internal pressure within the reactor, especially during the initial phase of the process. This was particularly evident in the first week of the test, when the reactor was purged three times, coinciding with peak biogas production. It is recommended to evaluate actions such as reducing the frequency of punctures, increasing headspace, and venting biogas at controlled intervals during batch tests, particularly in BPM assays. The device also demonstrated its advantage in real-time detection of potential issues related to methanogenesis inhibition.
